# Infective endocarditis caused by *Lactobacillus rhamnosus* in an immunocompetent patient without structural heart disease or invasive procedures: case report and literature review

**DOI:** 10.1093/ehjcr/ytaf645

**Published:** 2025-12-11

**Authors:** José Alejandro Claros Ruiz, Carlos Sánchez Sánchez, Ricardo Vivancos Delgado, Daniel Gaitán Román, Antonio José Plata Ciezar

**Affiliations:** Department of Cardiology, Hospital Regional Universitario de Málaga, Av. de Carlos Haya, 84, Málaga 29010, Spain; Department of Cardiology, Hospital Regional Universitario de Málaga, Av. de Carlos Haya, 84, Málaga 29010, Spain; Department of Cardiology, Section of Cardiac Imaging and Functional Testing, Hospital Regional Universitario de Málaga, Av. de Carlos Haya, 84, Málaga 29010, Spain; Department of Cardiology, Section of Cardiac Imaging and Functional Testing, Hospital Regional Universitario de Málaga, Av. de Carlos Haya, 84, Málaga 29010, Spain; Department of Infectious Diseases, Hospital Regional Universitario de Málaga, Av. de Carlos Haya, 84, Málaga 29010, Spain

**Keywords:** *Lactobacillus rhamnosus*, Endocarditis, Bacterial, Probiotics, Heart valve diseases, Echocardiography, Transoesophageal, Case report

## Abstract

**Background:**

Infective endocarditis caused by *Lactobacillus rhamnosus* is extremely rare, particularly in immunocompetent patients without structural heart disease or recent invasive procedures.

**Case summary:**

A 42-year-old immunocompetent man with no structural heart disease or recent interventions presented with fever and dyspnoea. Blood cultures grew *L. rhamnosus*. Transoesophageal echocardiography revealed a large vegetation on the aortic valve causing severe regurgitation, along with severe mitral regurgitation due to anterior leaflet perforation. The only identifiable risk factor was regular probiotic consumption. The patient underwent successful double valve replacement and completed 6 weeks of antibiotic therapy with favourable outcome.

**Discussion:**

A literature review identified 22 published cases of *L. rhamnosus* endocarditis. Most were associated with underlying valvular disease, invasive procedures, or probiotic use. This case highlights the need to consider *Lactobacillus spp.* as a potential pathogen, not merely a contaminant, in the appropriate clinical context, and calls for careful patient selection and monitoring when prescribing probiotics.

Learning points

- *Lactobacillus rhamnosus* should not be automatically considered a contaminant when isolated from blood cultures, especially in symptomatic patients.

## Introduction

*Lactobacillus rhamnosus* is a facultative anaerobic, gram-positive bacillus commonly found in the human gastrointestinal and genitourinary tracts and widely used in probiotic supplements. Although generally regarded as non-pathogenic, it has rarely been implicated in invasive infections such as infective endocarditis (IE). Reported cases of *L. rhamnosus* endocarditis typically occur in patients with structural heart disease, recent invasive procedures, or immunosuppression.^1,2^

We present a rare case of *L. rhamnosus* IE in an immunocompetent patient without structural heart disease or recent invasive interventions, in whom the only identifiable risk factor was regular probiotic use. This case underscores the importance of recognizing probiotic strains as potential pathogens in the appropriate clinical context and contributes to the limited body of literature addressing this emerging association.

## Summary figure

**Figure:**
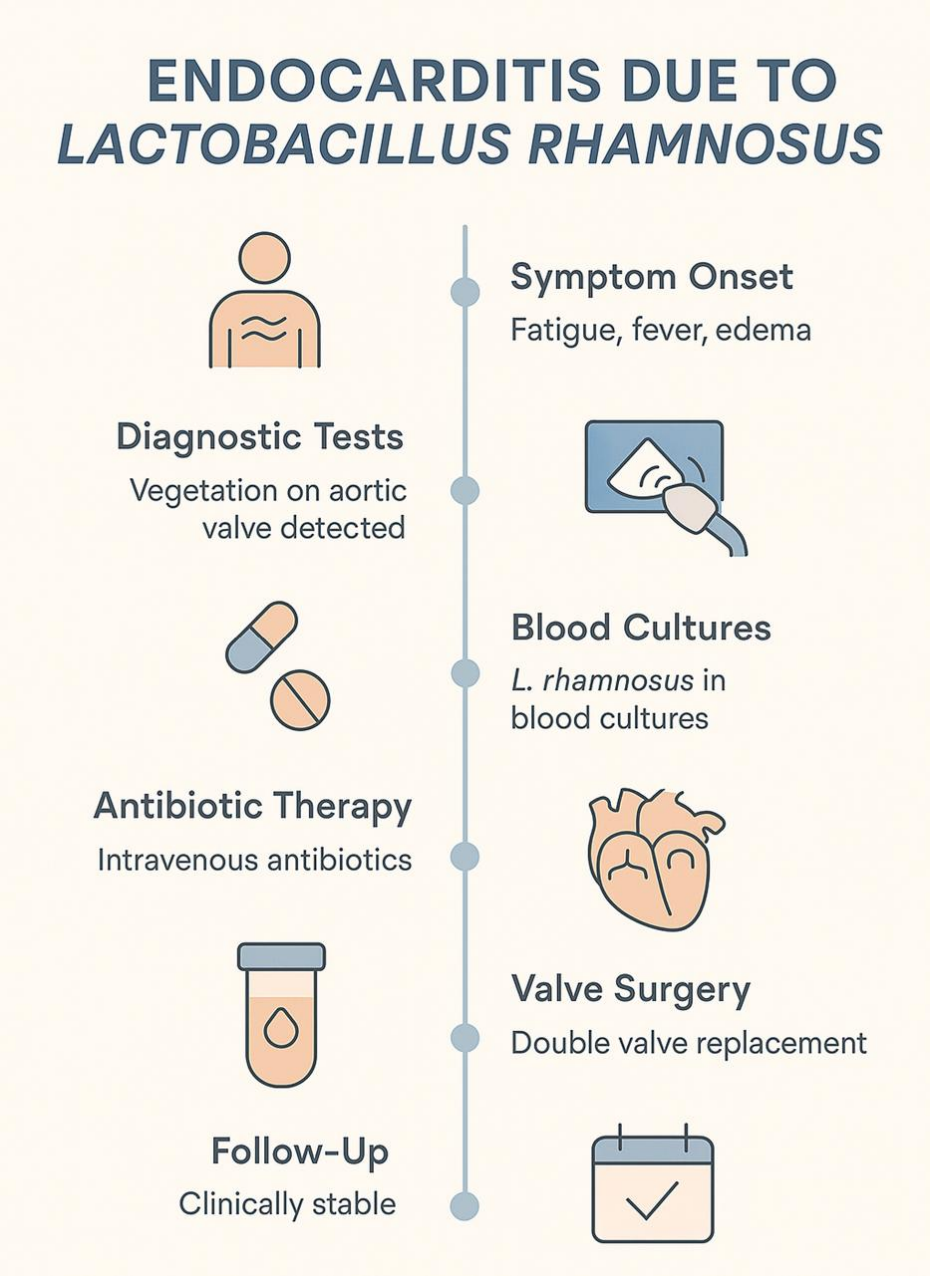


## Case presentation

A 42-year-old male with no relevant medical history presented to the emergency department with a 4-month history of progressive asthenia, bilateral lower limb oedema, intermittent evening fevers reaching 38.8°C, anorexia, and 16-kg weight loss. He had no known structural heart disease or immunosuppressive condition. He worked as a cook and reported active smoking (10–15 pack-years), occasional alcohol use (4–5 standard drinks/week), and recreational cocaine and prior cannabis use. He reported regular consumption of over-the-counter probiotic supplements. No recent dental or gastrointestinal procedures, animal contact, or high-risk exposures were identified.

Initial laboratory testing revealed normocytic anaemia (haemoglobin 10.3 g/dL; normal 13.5–17.5 g/dL), elevated C-reactive protein (92.9 mg/L; normal <5 mg/L), hyperferritinaemia (583 ng/mL; normal 30–400 ng/mL), polyclonal hypergammaglobulinaemia (immunoglobulin G 2060 mg/dL; normal 700–1600 mg/dL), and hypoalbuminaemia (2.6 g/dL; normal 3.5–5.0 g/dL). Peripheral smear showed microcytosis and occasional polychromasia. Serologies for Human Immunodeficiency Virus (HIV), hepatitis B/C, and autoimmunity were negative. Coombs direct test was weakly positive. Chest X-ray and abdominal ultrasound were unremarkable except for splenomegaly (16.7 cm). A blood culture taken in primary care 2 months prior had grown *L. rhamnosus*, initially dismissed as a contaminant. No antibiotic treatment had been initiated prior to admission, despite the positive outpatient blood cultures.

**Figure 1:**
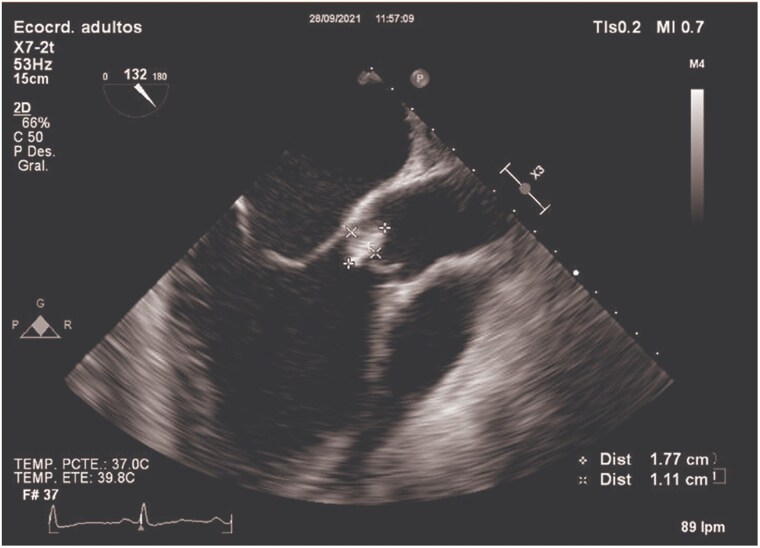
Transoesophageal echocardiography showing a 1.77 × 1.11 cm vegetation on the non-coronary cusp of the aortic valve.

**Figure 2:**
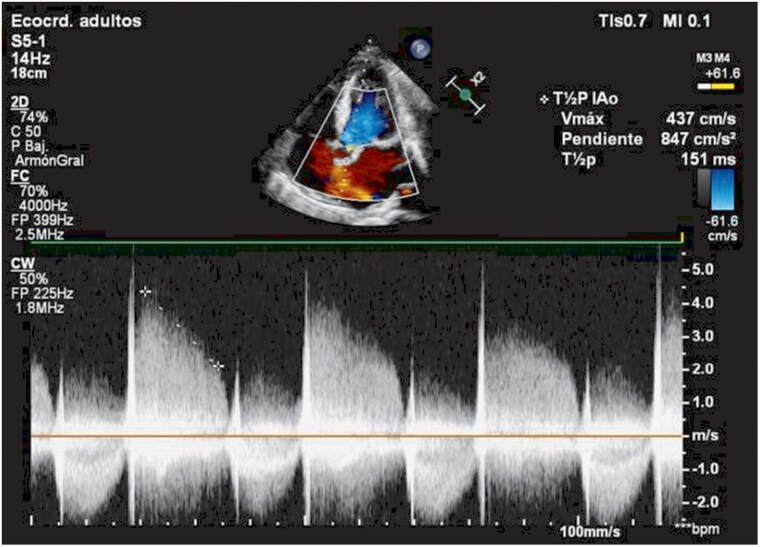
Transthoracic echocardiography with colour Doppler study showing multiple eccentric aortic regurgitation jets directed towards the anterior mitral leaflet, reaching the apex of the left ventricle. Pressure half-time was 151 ms.

**Figure 3:**
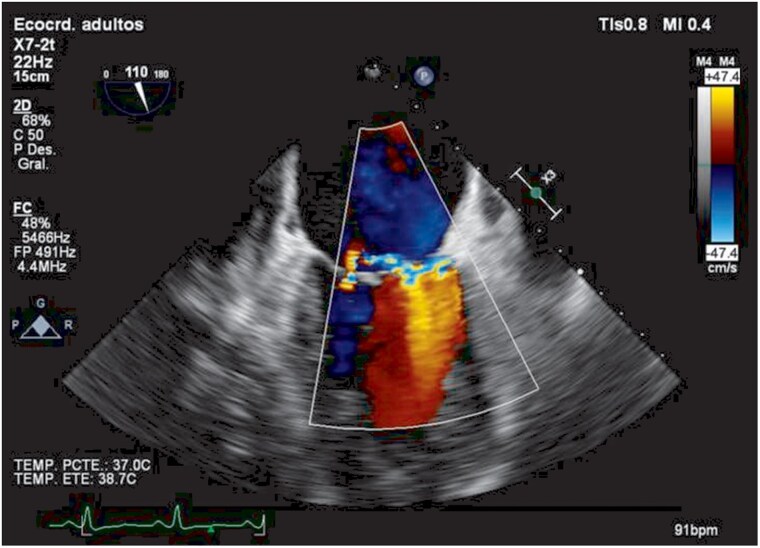
Transoesophageal echocardiography with colour Doppler showing anterior mitral leaflet perforation at segment A2 with a small regurgitant jet at the perforation site, together with a central jet of mitral regurgitation.

Physical examination showed mild malnutrition, pitting oedema to the knees, and a soft 1/6 systolic murmur at the apex. Repeat blood cultures again yielded *L. rhamnosus* in two separate sets. Transthoracic echocardiography showed severe aortic and mitral regurgitation with a globose left ventricle. Transoesophageal echocardiography revealed a large vegetation (1.77 × 1.11 cm) on the non-coronary cusp of the aortic valve (*Figure 1*, Supplementary material online, *Video S1*), multiple eccentric aortic regurgitation jets (*Figure 2*, Supplementary material online, *Video S2*), and a perforation of the anterior mitral leaflet (A2 segment) resulting in clinically significant global mitral regurgitation (*Figure 3*, Supplementary material online, *Video S3*).

The patient met the 2023 Duke-International Society for Cardiovascular Infectious Diseases (ISCVID) criteria for definite IE,^3^ fulfilling two major criteria (echocardiographic evidence of vegetations and two positive blood cultures with a compatible microorganism) and one minor criterion (fever >38°C).

Empirical intravenous therapy included cloxacillin, gentamicin, and ampicillin. Following confirmation of *L. rhamnosus*, the regimen was adjusted to ampicillin plus daptomycin. The patient underwent successful double mechanical valve replacement (aortic and mitral). Intraoperative inspection revealed vegetations on both valves, although valve cultures were sterile, likely due to prior antibiotic exposure.

Antibiotic susceptibility testing showed sensitivity to clindamycin, erythromycin, linezolid, penicillin, ampicillin, and daptomycin, with resistance to meropenem, imipenem, and vancomycin.

Ampicillin was discontinued postoperatively due to fluid overload, and daptomycin was continued for 21 days. Upon discharge, the patient completed an additional 7-day course of oral amoxicillin per his request.

Follow-up transthoracic and transoesophageal echocardiograms showed well-functioning prosthetic valves, with preserved global left ventricular function and normal transprosthetic gradients. The patient remained afebrile and clinically stable, with no complications or recurrent bacteraemia at the 6-month follow-up.

## Discussion

Although *Lactobacillus spp.* are usually dismissed as contaminants in blood cultures, their potential as invasive pathogens should not be overlooked, particularly in patients with compatible clinical presentations. *Lactobacillus rhamnosus* has been identified as the species most commonly implicated in such infections, albeit rarely.^2,3^

To date, 22 cases of *L. rhamnosus* endocarditis have been reported, including our patient.^2–13^ A summary of the clinical features, treatment, and outcomes of all reported cases, including the present one, is shown in *Table 1*. The majority of patients were male (77.3%) with a mean age of 57 years. Notably, none were immunocompromised. However, 72.7% had underlying structural heart disease, most commonly bicuspid aortic valve.^4,5^ Nearly half of the patients (45.5%) reported regular probiotic use, and 36.4% had undergone recent invasive procedures (dental or gastrointestinal interventions).^6^

**Table 1:** Summary of reported cases of *Lactobacillus rhamnosus* endocarditis

Characteristic	*N* (%)
Age (mean ± standard deviation)	56.59 ± 19.33
Sex (male/female)	17/5 (77.27%/22.73%)
Immunosuppression	0
Pre-existing valvular disease	16 (72.73%)
Invasive procedure	8 (36.4%)
Dental	4 (18.18%)
Gastrointestinal	3 (13.64%)
Nasal	1 (4.54%)
Probiotic use	10 (45.45%)
Symptoms	
Fever	14 (63.64%)
Fatigue	9 (40.91%)
Weight loss	8 (36.36%)
Dyspnoea	7 (31.82%)
Sweating	5 (22.73%)
Lower limb oedema	3 (13.64%)
Acute confusional syndrome	2 (9.09%)
Physical examination	
Murmur	17 (72.27%)
Arthritis	4 (18.18%)
Splinter haemorrhages	3 (13.64%)
Splenomegaly	2 (9.1%)
Janeway lesions	1 (4.54%)
Duration of symptoms before antibiotic initiation	
10 days	1 (4.54%)
30 days	4 (18.18%)
45 days	1 (4.54%)
90 days	2 (9.1%)
180 days	2 (9.1%)
270 days	1 (4.54%)
Unknown	11 (50%)
Mean ± SD	89.55 ± 84.51
Valve involved	
Aortic	9 (40.91%)
Mitral	7 (31.82%)
Aortic and mitral	4 (18.18%)
Unknown	2 (9.1%)
Surgical intervention required	15 (68.18%)
Death	4 (18.18%)
Antibiotic susceptibility	
Penicillin G	10 (45.45%)
Clindamycin	10 (45.45%)
Ampicillin	8 (36.36%)
Erythromycin	8 (36.36%)
Gentamicin	5 (22.73%)
Linezolid	3 (13.64%)
Rifampicin	3 (13.64%)
Daptomycin	3 (13.64%)
Ciprofloxacin	2 (9.09%)
Chloramphenicol	2 (9.09%)
Tetracycline	2 (9.09%)
Cefotaxime	2 (9.09%)
Amoxicillin/clavulanate	2 (9.09%)
Netilmicin	1 (4.54%)
Rifampicin	1 (4.54%)
Ceftriaxone	1 (4.54%)
Piperacillin	1 (4.54%)
Pristinamycin	1 (4.54%)
Levofloxacin	1 (4.54%)
Imipenem	1 (4.54%)
Unknown	5 (22.73%)
Antibiotic resistance	
Vancomycin	15 (63.64%)
Cefotaxime	3 (13.64%)
Trimethoprim/sulfamethoxazole	3 (13.64%)
Meropenem	3 (13.64%)
Imipenem	2 (9.1%)
Ceftriaxone	2 (9.1%)
Fluoroquinolones	1 (4.54%)
Cefazolin	1 (4.54%)
Cefaloridine	1 (4.54%)
Cefoxitin	1 (4.54%)
Gentamicin	1 (4.54%)
Methicillin	1 (4.54%)
Penicillin	1 (4.54%)
Rifampicin	1 (4.54%)
Fusidic acid	1 (4.54%)
Cotrimoxazole	1 (4.54%)
Unknown	5 (22.73%)
Number of antibiotics	
Monotherapy	4 (18.18%)
Dual therapy	10 (45.45%)
Triple therapy	4 (18.18%)
Quadruple therapy	1 (4.54%)
Unknown	3 (13.64%)
Antibiotic duration	
4 weeks	1 (4.54%)
6 weeks	9 (40.91%)
7 weeks	1 (4.54%)
8 weeks	2 (9.1%)
Unknown	9 (40.91%)
Comparison between Lactobacillus in blood culture and probiotic strain:	
Genetically identical	3 (13.64%)
Some differences	1 (4.54%)
Unknown	18 (81.82%)
Valve culture	
Positive	5 (22.73%)
Negative	7 (31.82%)
Unknown	10 (45.45%)

Summary of clinical characteristics, management, and microbiological findings in 22 reported cases of *Lactobacillus rhamnosus* infective endocarditis, including the present case. Data are presented as absolute values and percentages.

In contrast, our patient was immunocompetent, with no structural heart defects or recent procedures, making this case notable. The only identifiable risk factor was frequent over-the-counter probiotic use. While causality cannot be definitively proven, previous studies using molecular typing demonstrated genetic similarity between *L. rhamnosus* isolated from blood cultures and the corresponding probiotic strain in up to three cases.^3,7^ These findings suggest a potential pathogenic role for probiotics via bacterial translocation across disrupted mucosal barriers.

The diagnosis of *L. rhamnosus* endocarditis can be challenging, as the organism is often dismissed as a contaminant. However, in symptomatic patients with persistent bacteraemia, particularly in the presence of risk factors, *Lactobacillus* spp. should be considered true pathogens.^8^ Our case fulfilled the 2023 Duke-ISCVID criteria for definite IE.^1^

Treatment may be complicated by antimicrobial resistance. In our review, vancomycin resistance was reported in 63.6% of cases, and occasional resistance to carbapenems and cephalosporins was noted.^9^ However, susceptibility to penicillin, ampicillin, and clindamycin remains common.^10^ A dual-antibiotic regimen for 6 weeks was the most frequently used approach. Surgical intervention was necessary in 68.2% of cases, often due to valve destruction or heart failure.^11,12^

Our case underscores the need for vigilance in interpreting blood cultures, particularly in symptomatic patients, and reinforces the importance of thorough clinical evaluation, including dietary habits and probiotic use. With the increasing popularity of probiotic supplementation, physicians should carefully assess the risk–benefit ratio, especially in patients with structural heart disease or prosthetic valves. Regulatory agencies have acknowledged the lack of safety data regarding probiotics in these populations.^13^

## Conclusions

This case highlights that *L. rhamnosus*, although generally regarded as non-pathogenic, can cause severe IE even in immunocompetent individuals without structural heart disease or recent invasive procedures. Clinicians should avoid dismissing *Lactobacillus* spp. in blood cultures as contaminants, especially in symptomatic patients, and should consider the potential pathogenic role of probiotics. Careful clinical evaluation, including dietary history and appropriate imaging, is essential for early diagnosis and management. Further studies are warranted to better define the safety profile of probiotics in at-risk populations.

## Lead author biography


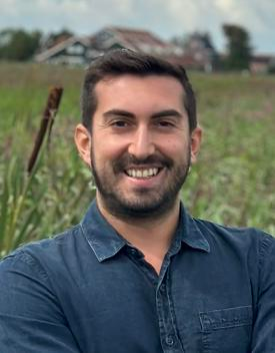


José Alejandro Claros Ruiz is a cardiology resident at the Hospital Regional Universitario de Málaga (Spain). He is currently undergoing clinical training in advanced cardiac imaging and heart failure. His main areas of interest include infective endocarditis, valvular heart disease, and the integration of imaging in complex clinical decision-making.

## Supplementary Material

ytaf645_Supplementary_Data

## Data Availability

The data underlying this article are available in the article itself. Further enquiries can be directed to the corresponding author.
